# REDD implementation for greenhouse gas reduction and climate change mitigation in Hyrcanian forests: a case study of the Kojoor Watershed, Northern Iran

**DOI:** 10.1007/s10661-024-12616-z

**Published:** 2024-04-25

**Authors:** Arman Niknam, Reza Sarli, Mehrnoosh Taherizadeh, Sina Attarroshan, Fatemeh Pourmansouri

**Affiliations:** 1 Department of Geoinformatics Physical and Environmental Geography, University of Szeged, Szeged, Hungary; 2 Department of Forest Resources Management, Faculty of Forestry, University of Agriculture in Krakow, Al. 29 Listopada 46, 31-425 Krakow, Poland; 3 Environment Department, Islamic Azad University, Ahvaz Branch, Ahvaz, Iran; 4 Department of Water Resources Engineering and Management, Faculty of Engineering, K. N, Toosi University of Technology, Tehran, Iran

**Keywords:** BioCF, Deforestation, Forest conservation, CO2 reduction, Sustainability

## Abstract

Reducing emissions from deforestation and forest degradation (REDD) is a specific strategy for combating deforestation and forest degradation to alleviate the effects of climate change. In this study, the potential greenhouse gas (GHG) emission reduction resulting from the implementation of a REDD project is estimated. Changes in forest cover throughout the years 1985, 1990, 1995, 2000, 2010, 2015, and 2020 were analyzed using time-series Landsat imagery (TM, ETM + , and OLI) and a random forest algorithm. Multilayer perceptron neural networks were used to model the transition potential of the forest cover, which were then predicted via Markov chain analysis. The change detection analysis revealed two discernible patterns in forest cover dynamics. Between 1985 and 2000, a notable decrease in forest cover was seen, whereas from 2000 to 2020, it significantly increased. The results suggested that the absence of REDD implementation would result in the deforestation of approximately 199,569 hectares of forest cover between 2020 and 2050, leading to the release of 1,995,695 tCO2e of emissions into the atmosphere. However, with the implementation of REDD, these emissions would be reduced to 405,512 tCO2e, effectively preventing the release of 1,590,183 tCO2e of emissions into the upper atmosphere. This study demonstrates that the implementation of REDD projects can be an effective strategy for reducing GHG emissions and mitigating climate change in the Hyrcanian forests.

## Introduction

Global warming is a significant concern for the international community due to its far-reaching implications on the environment and the socioeconomic progress of societies (Brown et al., 2021; Haruna et al., 2023). Climate warming can be attributed to multiple significant factors, including the emission of greenhouse gases (GHGs), burning of fossil fuels, deforestation, forest degradation, industrial processes, agricultural activities, and land-use changes (Roshan et al., 2021). Forests play a vital role as essential ecosystems offering crucial goods and services. They regulate and safeguard the quality of air, soil, and water while reducing the concentration of GHGs and promoting climate stability (Howe et al., 2019; Weishou et al., 2011). The impact of deforestation and forest degradation on global warming is undeniable (Savaresi, 2016). This disrupts the Earth’s energy balance due to the loss of carbon sequestration capacity, leading to increased surface temperatures and regional warming (Cadman et al., 2019; Corbera, 2012). Furthermore, deforestation or the degradation of forests leads to the release of carbon dioxide (CO2) into the atmosphere, heightening the greenhouse effect (Savaresi, 2016). The increased CO2 exacerbates the natural greenhouse effect, contributing to the general warming of the Earth’s climate. This, in consequence, disrupts intricate feedback loops, triggering a cascade of effects on climate systems. Additionally, deforestation contributes to the loss of biodiversity and habitat fragmentation, directly impacting ecosystem functions and carbon cycling. It is evident that swift action is necessary to address deforestation and its impact on our planet (Beygi Heidarlou et al., 2023). Deforestation and land-use/land-cover (LULC) changes in developing nations that are necessary for timber production and agricultural expansion are the primary sources of GHG emissions. The conversion of forested areas into non-forested areas in developing countries significantly affects the accumulation of GHGs in the atmosphere (Olander et al., 2008). Factors such as LULC changes, illegal logging (Zeb et al., 2019), insect outbreaks (van Lierop et al., 2015), wildfires (Nasiri et al., 2022a), bad management (Muttaqin et al., 2019), overgrazing (Niu et al., 2021), and other disturbances contribute to forest loss and degradation. These factors hinder the regeneration and recovery of forests, leading to their decline and loss (Achard et al., 2012). Deforestation and LULC changes are responsible for around 20% of global GHG emissions, exceeding the emissions of the transportation sector (Olander et al., 2008).

Deforestation and forest degradation are major contributors to the loss of carbon sinks, biodiversity, and sustainable land management practices. To combat this, it is vital to accurately and systematically monitor deforestation and forest degradation to gain essential data on the extent, causes, and patterns of forest loss. This information serves as a foundation for informed decision-making and the development of targeted strategies to mitigate the adverse impacts of climate change (Ghanbari et al., 2018; Leon et al., 2022). The reducing emissions from deforestation and forest degradation (REDD) strategy is one such targeted approach that aims to address deforestation and forest degradation to mitigate climate change (Muttaqin et al., 2019). Specifically, REDD is a scientific strategy that aims to provide financial incentives to developing countries to mitigate GHG emissions resulting from deforestation and forest degradation. The strategy acknowledges the crucial role of forests in sequestering CO_2_ from the atmosphere and incentivizes countries to conserve and sustainably manage their forest resources. Within the REDD framework, countries that successfully reduce deforestation and forest degradation will receive financial compensation for their prevented or reduced carbon emissions (Pattanayak et al., 2010). These incentives can originate from diverse sources, such as international funds, carbon markets, and bilateral agreements. Importantly, the REDD strategy extends beyond addressing deforestation and forest degradation alone. It encompasses additional elements, including forest conservation, sustainable forest management, enhancement of forest carbon stocks, and ensuring the rights and livelihoods of local communities (RRI, 2014; Gilmour, 2016; Muttaqin et al., 2019).

The results of the REDD scenario should be examined for an accurate understanding of the natural phenomena in the environment (Gaveau et al., 2013; Parker et al., 2008; Zimmerman & Kormos, 2012). In this regard, various researchers have used remote sensing datasets and tools based on geographic information systems to explore the impact of deforestation prevention on GHGs emissions and carbon storage. Ty et al. (2011) in Cambodia researched and simulated forest cover changes. They claimed the REDD + initiative might save 8.6 million tons of CO2 from being released. Further, a study conducted in Nigeria during 1990–2009 evaluated the severity of deforestation. The simulation of the REDD + scenario for the year 2040 revealed that under the conservation scenario, the storage of 1,606,147 tCO2e and prevention of its emission could be achieved (Bununu et al., 2016). Similarly, a study conducted in Indonesia assessed the emission of greenhouse gases resulting from deforestation. The simulation results indicated that the implementation of REDD + could decrease carbon emissions from 26 million tCO2e to 18 million tCO2e (Nahib & Suwarno, 2018). Another study by Parsamehr et al. (2019) evaluated changes in forest cover and carbon reserves in certain parts of Iran. The results of the study suggested that 705,336 tCO2e would be emitted into the atmosphere by 2044 under the baseline scenario. This literature review reveals the complexity of the relationship between deforestation, GHG emissions, and global warming, with variations observed across different regions. Deforestation trends in various parts of the world have been extensively studied. However, a notable research gap exists concerning the Hyrcanian forests, a unique region whose specific dynamics and deforestation effects warrant investigation.

Temperate deciduous forests are scarce and limited in the arid and semi-arid Middle East. The Hyrcanian ecoregion, situated on the southern coast of the Caspian Sea in northern Iran, is the most notable area where these forests can be found (Gholizadeh et al., 2020). The Hyrcanian forests, spanning over an area of approximately 55,000 km2 along the southern coast of the Caspian Sea, provide a wide range of ecosystem services. These services include climate regulation, human health benefits, wildlife habitats, tourism and recreation opportunities, nutrient cycling, erosion control, biodiversity conservation, disturbance regulation, and freshwater supply (Asadolahi et al., 2017). The Hyrcanian forests are known for their Arcto-Tertiary relict elements, which hold significant phytogeographical importance. But, due to the rising human activities in this region, the conservation of these forests has become a major concern. As a result, in 2019, the Hyrcanian forests were recognized as a UNESCO World Heritage property, emphasizing the need to protect and preserve this threatened forest zone (Ghorbanalizadeh & Akhani, 2022).

In short, the intricate relationship between deforestation, greenhouse gas emissions, and global warming demands urgent attention and decisive action. As we navigate the challenges posed by deforestation globally, it is essential to extend our focus to unique ecosystems like the Hyrcanian forests, recognizing their ecological significance and the need for preservation. Embracing strategies such as REDD provides a pathway toward mitigating climate change by addressing the root causes of deforestation. In this regard, this study aims to address the mitigation of global warming by focusing on GHG emission reduction. Specifically, the potential impact of the implementation of a REDD project within the protected area of Kojoor, located in the Hyrcanian forests, north Iran, is modeled. The effectiveness of the REDD framework in reducing GHG emissions in the Hyrcanian forests is assessed. Through the visualization of areas at risk of substantial deforestation, habitat fragmentation, and carbon stock depletion by 2050, our scientific investigation provides valuable insights for decision-making processes and future land-use planning, particularly in relation to the preservation of the Hyrcanian forests, which hold significant world heritage value.

## Material and methods

### Study area

**Fig. 1:**
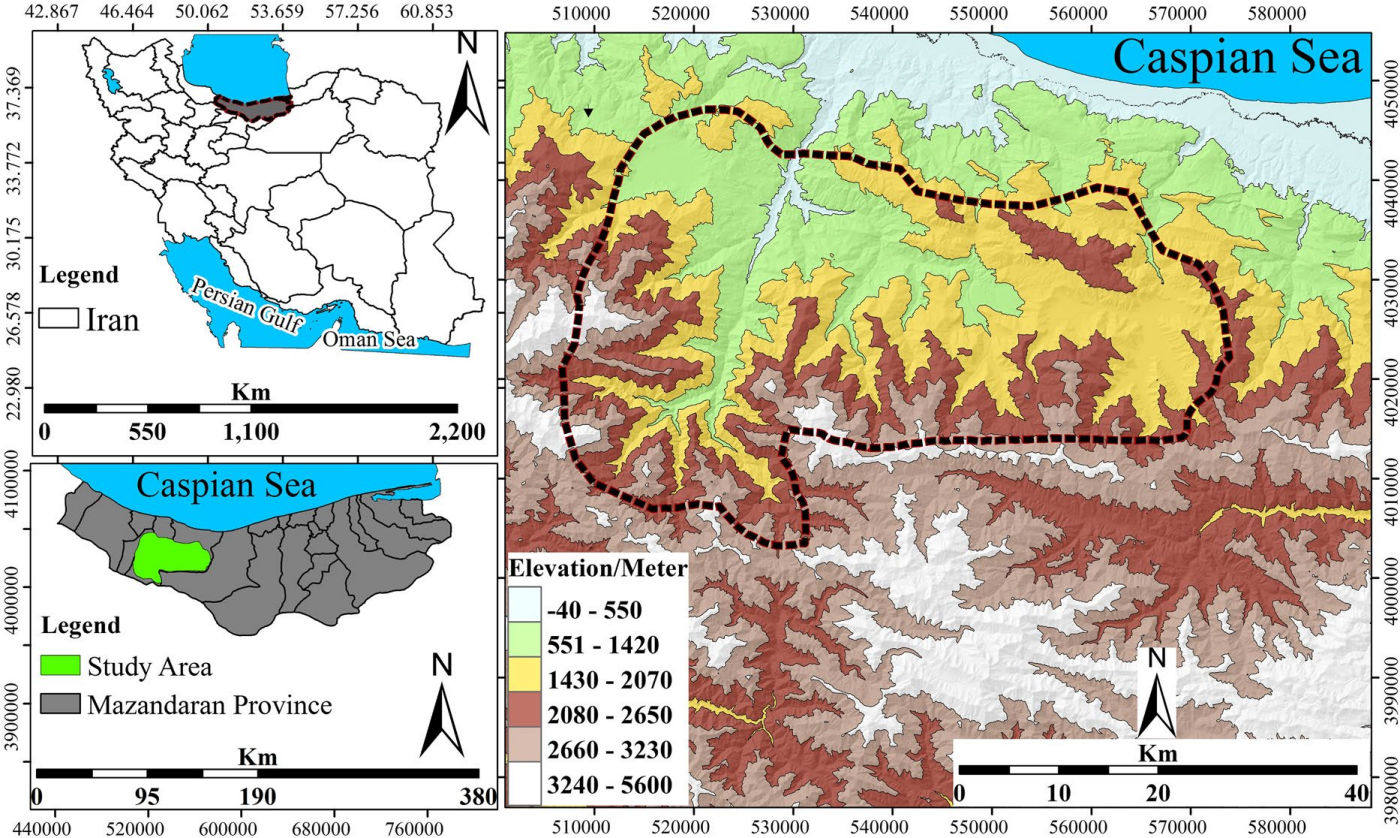
Geographical location of the study area

This study was conducted within the Kojoor watershed (Fig. 1), a protected area in the Hyrcanian forests, north Iran. The study site is notable for its diverse topography, elevated altitude, and humid subtropical climate, which exhibits distinct seasonal variations (Fig. 1). The altitude of the area ranges from 432 to 3800 m (2002 m on average) above sea level. The region receives an annual average precipitation of approximately 869 mm; the maximum value is observed in October, and the minimum is in February. The landscape mostly consists of the Hyrcanian forests; some parts are pastures and agricultural lands. The Hyrcanian forests, a World Heritage Site, are mainly covered by deciduous trees, such as common hornbeam, oak, Persian ironwood, and Oriental beech (Nasiri et al., 2023a, b). The Hyrcanian forests are also home to a variety of wildlife, including bears, leopards, wolves, wild boars, and different types of birds. These forests provide habitats and ecosystems for these animals and many plant species (Darvishsefat, 2006). The Central Alborz Protected Area, which has an area of ​​399,000 ha and is one of the most valuable regions of Iran in terms of gene and species diversity, was added to a list of protected areas in the world after the approval of the Supreme Environmental Council of Iran in 1967. The existence of four forest reserves in the protected area of ​​Kojoor is another sign of the value of the forests in this region, especially for the protection of *Cupressus sempervirens*, *Buxus hyrcanus*, and *Alnus glutinosa* (Meteorological Organization of Iran, n.d.).

### Remote sensing datasets and LULC maps

Accurate LULC maps are the first component of such studies of deforestation (Nasiri et al., 2022b). This requires remote sensing, which can interpret massive amounts of spatial and temporal data (Mansourihanis et al., 2023; Taherizadeh et al., 2023a, b). Based on our research objectives and methodology, LULC maps were used to (1) create an accurate forest/non-forest mask to model the effect of REDD implementation and (2) map the driving forces of LULCCs. We used the capabilities of the Google Earth Engine cloud computing platform to process multisensor Landsat data. As the primary step in LULC mapping, a multitemporal reference dataset, including training and validation samples, was generated based on in situ recording using the Global Positioning System and the visual interpretation of aerial and very-high-resolution (VHR) Google Earth images. Field surveys were performed in the summer of 2020 to collect precise samples, which were grouped in classes with a high level of spectral similarity (Table 1); for the other time points, these samples were overlaid with the abovementioned VHR images, and a revision process was conducted. For each time point (1985, 1990, 1995, 2000, 2010, 2015, and 2020), a total of 800 samples representing six LULC classes were collected: forests, rangelands, croplands, barren lands, artificial lands, and water bodies. The samples in the reference dataset were randomly divided into training (70%) and testing (30%) subsets.

**Table 1:** The $characteristics$ of the reference dataset for the year 2020 (pixel size = 30 m)

LULC	Training samples		Validation samples	
	Sample no	Pixel no	Sample no	Pixel no
Forest	154	1386	66	594
Rangeland	98	882	42	378
Cropland	98	882	42	378
Barren lands	63	567	27	243
Artificial lands	84	756	36	324
Water bodies	63	567	27	243

As for the classification features, at each time point, we created an image collection based on all the Landsat images captured during the growing season (from April to September) and calculated pixel-wise spectral–temporal metrics (STMs) based on the resulting image collections. In addition to the spectral bands, common vegetation indices, namely, the difference vegetation index (DVI), normalized DVI (NDVI), and enhanced vegetation index, were used to distinguish different vegetation types and perform LULC mapping (Kordi & Yousefi, 2022; Rahmanian et al., 2023). Finally, STMs were calculated as the median, standard deviation, minimum, maximum, and percentile (25th, 50th, and 75th) values for all bands and vegetation indices (Table 2). For further analysis, the LULC map was classified into two distinct classes: forest and non-forest.

**Table 2:** Details of image collections and features used for LULC mapping

	Sensor	Image no	Features (STMs)	Feature No
1985	Landsat-5, TM	10	Median, standard deviation, minimum, maximum, and percentiles (25th, 50th, and 75th)	63
1990	Landsat-5, TM	19		
1995	Landsat-5, TM	27		
2000	Landsat-7, ETM +	14		
2010	Landsat-5, TM	25		
2015	Landsat-8, OLI	22		
2020	Landsat-8, OLI	28		

The training samples (Table 1) and STMs (Table 2) were used to train a random forest (RF) model for LULC classification. The RF model, rooted in decision trees, demonstrates exceptional performance in analogous studies owing to its ability to effectively differentiate and map classes sharing similar spectral attributes (Chang et al., 2020; Nasiri et al., 2023a, b; Yin et al., 2021). Unlike alternative methods, RF demands less computational time, has fewer parameters to tune, and exhibits adeptness in managing multi-modal data. The effectiveness of the RF model correlates with its hyperparameters employed during training. These parameters can be fine tuned to enhance the learning process and generate more precise models. In this study, we utilized the GEE-based hyperparameter optimizer to ascertain the optimal value for “ntree” (the number of trees). The alteration in accuracy assessment served as a criterion for determining this parameter. Ultimately, “ntree” was set to 500 for model training and classification.

### Change detection, transition potential, and LULC prediction

During the study period, the multitemporal LULC maps were used to identify and track deforested areas. Markov chain analysis was conducted to predict future LULCCs, and the predictions were subsequently utilized as input data to analyze GHG emissions under the REDD project in the coming decades. The probability of changing the land cover of a system into a situation (changing the land cover of a system at a particular time) at a particular time (${t}_{2}$) was obtained from information about the same system in the past (${t}_{1}$) and did not depend on information from times before ${t}_{1}$, as such a transition is a one-time process (Eq. 1). The primary assumption in Markov chain analysis is that future land-use changes are primarily influenced by past trends or patterns in land-use changes. This assumption is rooted in the idea that the transition probabilities between land-use categories remain consistent over time based on historical observations. Essentially, the model assumes that the probabilities of transitioning from one land-use class to another are determined by the historical sequence of changes. This assumption of persistence or dependency on past trends forms the core principle of the Markov chain model in this study and guides our predictive capability. In the Markov model, the LULCC at ${t}_{2}$ was obtained using its distribution at ${t}_{1}$ by calculating the transition matrix. The Markov chain is expressed as Eq. (2) (Eastman, 2003; Rajitha et al., 2010).

1 $${vt}_{2}={Mvt}_{1}$$

In this equation, ${vt}_{1}$ is the land cover vector at ${t}_{1}$, ${vt}_{2}$ is the land cover vector at ${t}_{2}$, and $M$ is the transition probability matrix $m\times m$ for the time interval $\Delta t={t}_{2}-{t}_{1}$.

The probability of transition between two states ($Pij$) is calculated using Eq. (3).

2 $$Pij= \frac{{n}_{ij}}{{n}_{i}},$$

where $${n}_{i}$$ is

3 $${n}_{i}= \sum_{j=1}^{q}{n}_{ij}.$$

Here, ${n}_{ij}$ is the number of pixels of class $i$ at ${t}_{1}$ that are changed into pixels of class $j$ at ${t}_{2}$ and $q$ is the total number of classes.

The Markov chain had three outputs. The model generated a transition probability matrix and a transition area matrix to show the probability of class changes and the number of pixels that may be converted into pixels of other classes. The model also generated a set of status probability images to indicate the position of each landcover (Fan et al., 2008; Pontius, 2000). Various biophysical variables were considered: elevation; slope; aspect; and distance from forests, rangelands, croplands, artificial lands, water bodies, and roads. The elevation, slope, and aspect layers were generated based on the shuttle radar topography mission digital elevation model. The distance between LULC classes was calculated based on the multitemporal LULC maps, and the distance between roads was calculated based on a road map obtained from the National Cartographic Center of Iran. The artificial neural network (ANN) model utilized actual changed pixels from the last time point for both training and testing data (dependent variables) (Roy et al., 2022; Ding et al., 2023; Mokayed et al., 2023; Preethi & Mamatha, 2023). The driving forces mentioned earlier served as explanatory variables (independent variables). This approach led to an iterative model calibration process, where the training data enabled the model to estimate change potential in areas without samples. The model computed training RMSE and testing RMSE errors, enabling accurate monitoring of the calibration process. Lower testing errors during iterations signified effective model training, ensuring its readiness for predictive use. Additionally, setting stopping criteria, including RMS = 0.01 and iterations = 1000, was crucial for successful model calibration.

For the prediction of future LULCCs, we used historical LULC data and transition probability maps and performed Markov chain analysis to simulate LULC maps for two time points: 2010 (based on LULCCs observed from 1985 to 2000) and 2020 (based on LULCCs observed from 2000 to 2010). To assess the accuracy of our simulations, we compared the simulated LULC maps with the classified LULC maps (“Remote sensing datasets and LULC maps” section) using three agreement metrics, namely, ${K}_{no}$, ${K}_{location}$, and ${K}_{quantity}$ (Eqs. 4–6).

4 $$\text{Kno } = \frac{{\text{P}}_{0}-{\text{NQNL}}}{{1}-{\text{NQNL}}}$$

5 $$\text{Klocation } = \frac{{\text{P}}_{0}-{\text{MQNL}}}{{\text{MQPL}}-{\text{MQNL}}}$$

6 $$\text{Kquantity } = \frac{{\text{P}}_{0}-{\text{NQML}}}{{\text{PQML}}-{\text{NQML}}}$$

The observed proportion correct, denoted as ${\text{P}}_{0}$, measures the accuracy of the results. $NQNL$ represents the expected proportion correct in cases without quantity and location information. $MQNL$ indicates the percentage of correct outcomes when there is a moderate ability to specify the quantity but no ability to specify the location. $MQPL$ represents the percentage of correct outcomes when there is a moderate ability to specify the quantity and a perfect ability to specify the location. $NQML$ indicates the percentage of correct outcomes when there is no ability to specify the quantity and a moderate ability to specify the location (for additional details, see Pontius, 2000). Following the accuracy assessment of the simulated LULC maps, we used all observed changes to forecast LULCCs up to 2050.

### REDD scenario modeling based on BioCF approach

**Fig. 2:**
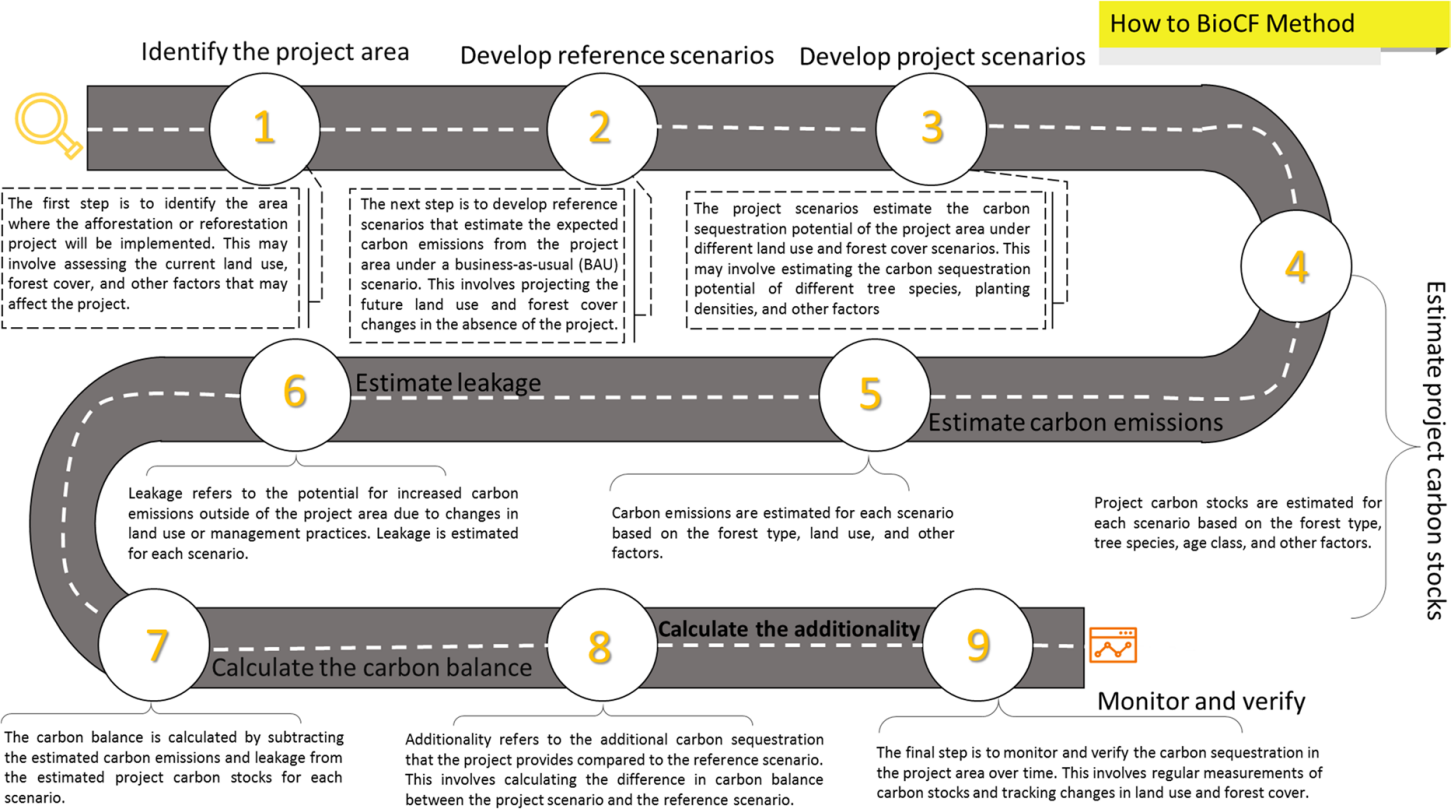
REDD scenario modeling based on the proposed BioCF method (Fund, 2008)

The BioCF method, proposed by the BioCarbon Fund in 2008, provides a framework for estimating the carbon sequestration potential of deforestation and reforestation projects under the REDD framework. This method involves a scenario modeling approach that considers various factors that influence carbon sequestration in forested areas. To establish the REDD baseline, it is crucial to delineate the project area and leakage belt. The project coverage is divided into the project implementation area for forest protection and the leakage belt where deforestation occurs. The objective within the project area is to counteract factors leading to forest destruction, thereby inhibiting their spread to the leakage belt. This approach aims to prevent deforestation over time in regions with substantial forest coverage that are vulnerable to degradation. Figure 2 shows the REDD scenario modeling process based on the BioCF method (Fund, 2008). The studied REDD project followed a 30-year projection, incorporating periodic assessments every 5 years. Carbon reporting was conducted based on these specific time intervals. The GHG emission reduction was calculated by subtracting the estimated carbon savings achieved through the REDD project intervention, including the estimated carbon losses resulting from leakage, from the estimated carbon loss that would occur without the REDD project(Ramachandra & Setturu, 2020). This carbon loss was specifically associated with LULCCs. The formula used for this calculation is

7 $$C-{\text{REDD}}=\left(C-{\text{Baseline}}\right)-\left(C-{\text{Actual}}\right)-\left(C-{\text{Leakage}}\right).$$

$C-Baseline$ represents the GHG emissions within the project area, $C-Actual$ represents the actual GHG emissions observed in the same area, $C-Leakage$ represents the GHG emissions resulting from leakage, and $C-REDD$ represents the net reduction in anthropogenic GHG emissions attributed to the $REDD$ project. All measurements are expressed in metric tons of CO_2_ equivalent (tCO2e).

## Results

### Accuracy assessment of LULC maps

The accuracy assessment of the LULC maps shows that the generated features and RF model can produce reliable sources for further analysis. The overall accuracy and kappa coefficient for all time points range from 87.78 to 92% and from 0.8 to 0.86, respectively. Per class, the F1 score for forests ranges from 92.52 to 94.31, indicating that the forest cover is mapped with high accuracy.

### Change detection analysis

**Fig. 3:**
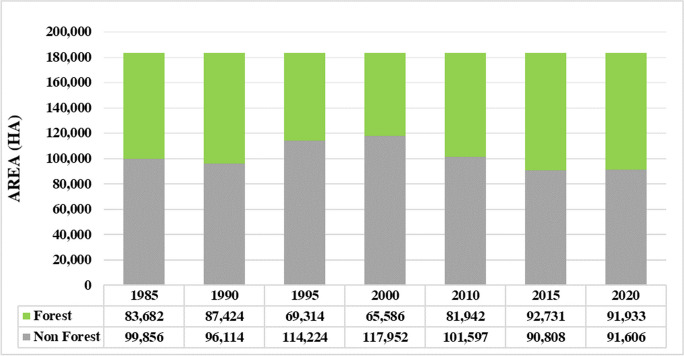
Forest cover dynamics (area) during the during the study time period

**Fig. 4:**
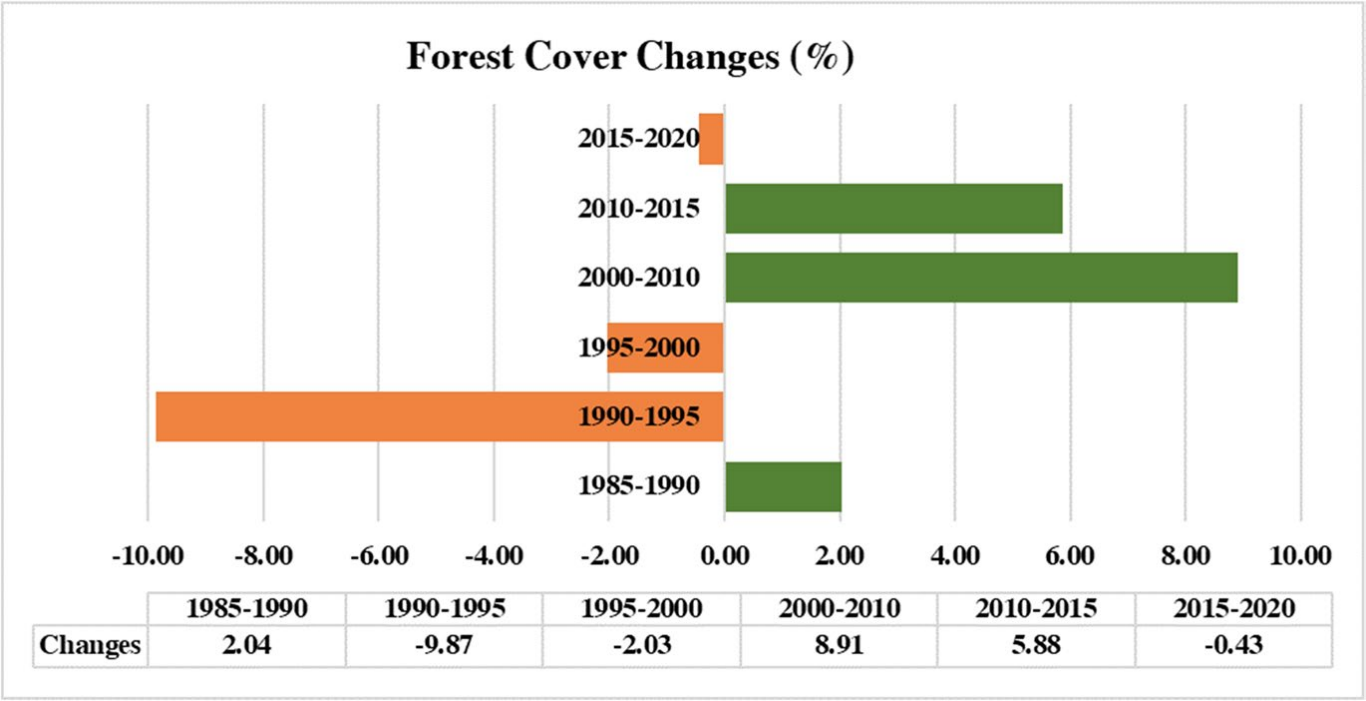
Changes in forest cover at different time points

The forest area in the study area was calculated based on the multitemporal LULC maps (Figs. 3  and 4). The result shows that the forest area ranges between 65,586 and 92,731 ha (all values rounded to hectare). Moreover, the change detection results reveal two general trends in forest cover changes: a decline from 1985 to 2000 and an increase from 2000 to 2020. The highest forest recovery (8.91%) occurs between 2000 and 2010. By contrast, the highest deforestation (− 9.87%) appears in 1990–1995. In general, during the study period (1985–2020), the forest area increases by 8251 ha.

### Projected LULC maps

**Fig. 5:**
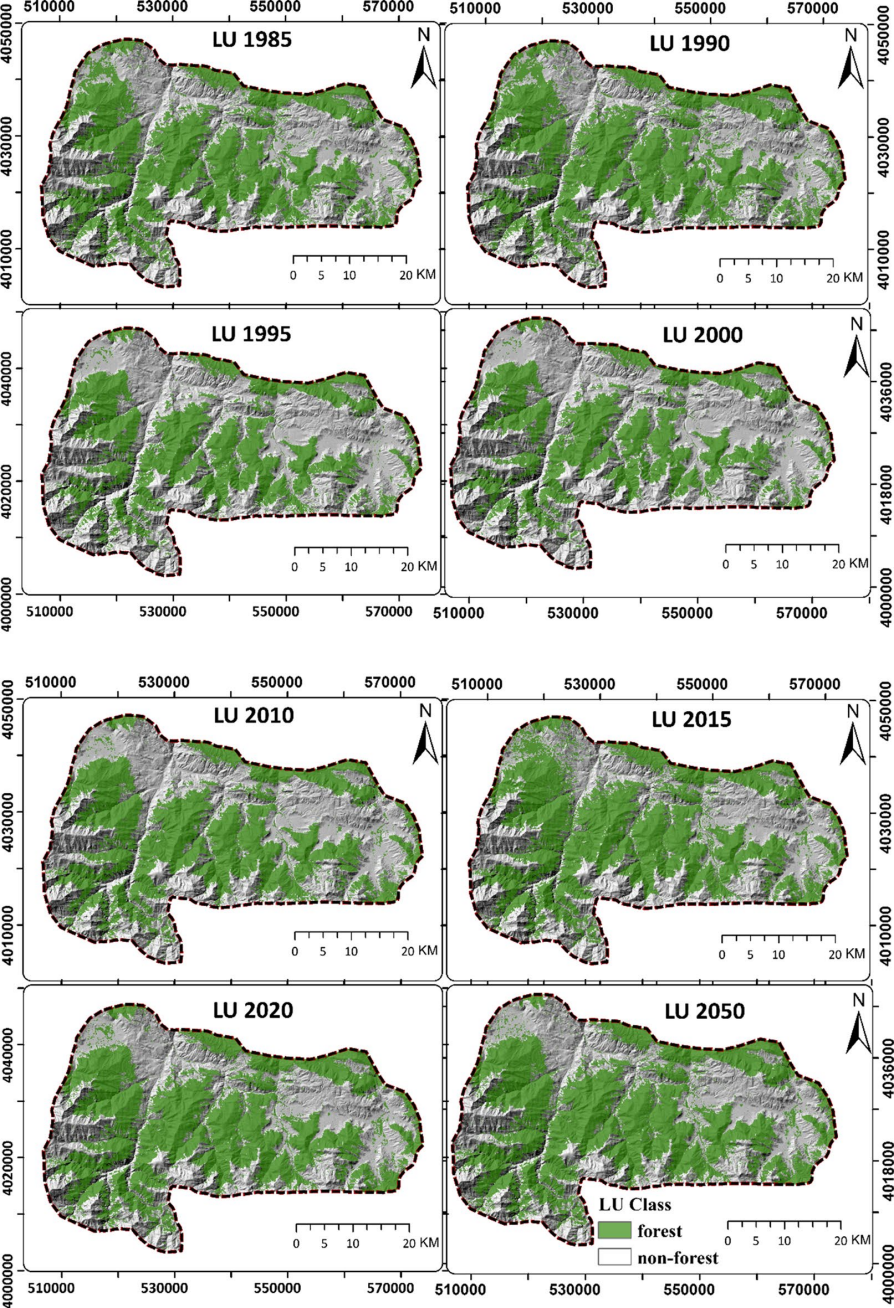
Land use plan envisaged in the REDD project area

The projected LULC maps based on the MLP and Markov chain analysis are presented in Fig. 5. Considering the change trajectories of the forest cover between 1985 and 2020 and based on the variables used for transition potential modeling, the forest area in the study area is expected to expand. Table 3 displays the accuracy assessment results of the projected LULC maps. The models perform strongly in predicting forest covers based on past changes. The Klocation metric ranges between 0.956 and 0.971, indicating a high level of accuracy in locating forested areas. Similarly, the Kquantity metric ranges between 0.980 and 0.993, demonstrating precise estimation of the forest cover quantity. These results signify the effectiveness of the models in accurately predicting forest covers based on historical data.

**Table 3:** Accuracy assessment of predicted forest cover maps

Simulate forest covermap	Kno	Klocation	Kquantity
2010	0.994	0.971	0.993
2020	0.993	0.956	0.980

### Scenario modeling

**Fig. 6:**
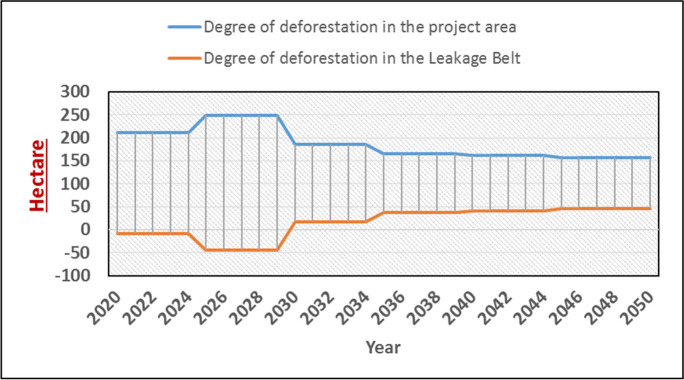
The rate of deforestation in the leakage belt and the project area under the BioCF methodology per year per hectare

According to Fig. 6, the long-term average (2020 to 2050) deforestation rates in the leakage belt (the “leakage belt” typically refers to the area surrounding a conservation or deforestation prevention project site) and the project area (the “project area” is the specific geographic region or location where a conservation or deforestation prevention project is implemented) are 15.12 and 188.08 ha per year, respectively, but this trend shows short-term changes. The average deforestation amounts for 2020–2022 are − 8.28 and 211.48 ha for the leakage belt and the project area, respectively. From 2026 to 2032, the averages are 17.42 and 185.77 ha for the leakage belt and the project area, respectively. Finally, from 2034 to 2050, these values reach 46.60 and 156.60 ha, respectively.

**Fig. 7:**
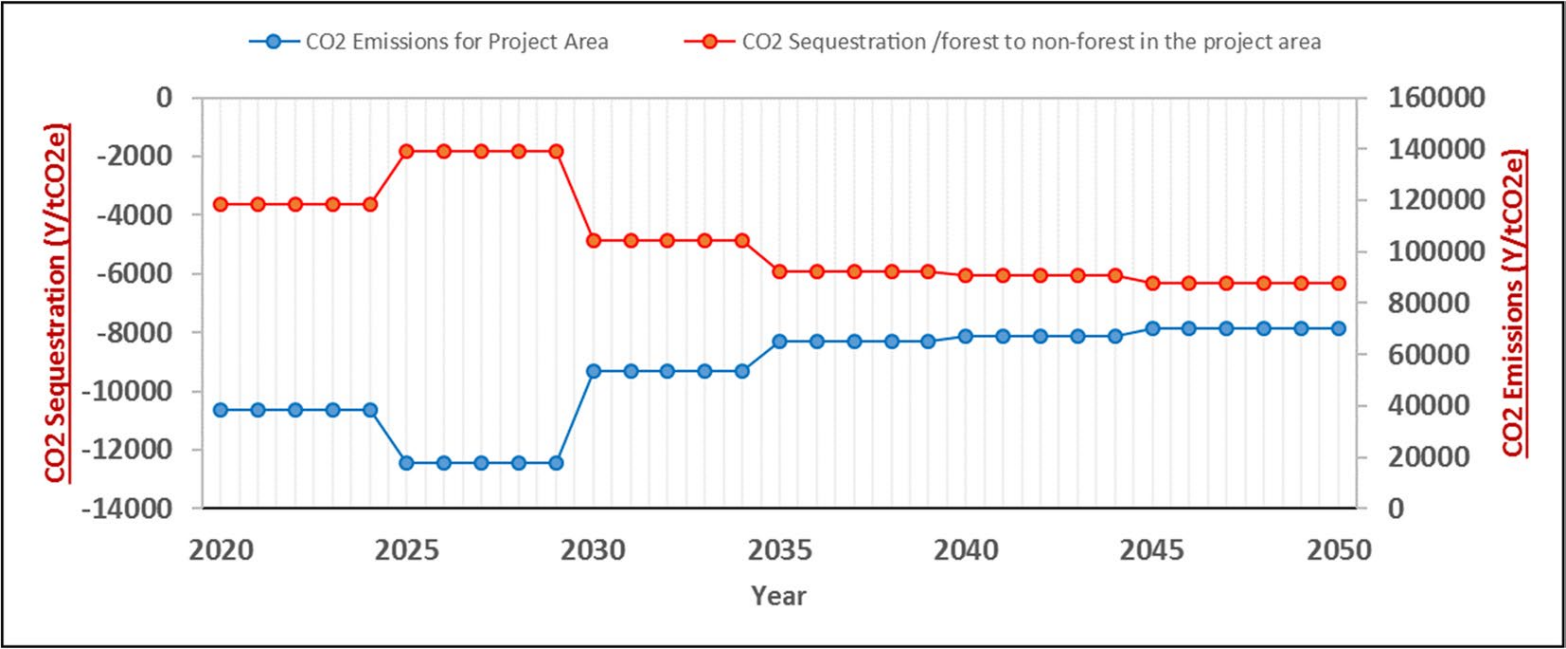
Diagram of CO2 emissions and carbon sequestration in the leakage belt under BioCF methodology per year in terms of ton

According to Fig. 7, the 30-year average amount of carbon sequestration due to the conversion of forests into non-forest areas in the project area is approximately 105.254 thousand tons, and the 30-year average amount of CO_2_ emissions in the project area is approximately − 9.449 tons. Over the 30 years, the changes show a discernible pattern. During the initial 5 years of project implementation, the conversion of forests into non-forest areas results in 118.655 thousand tons of carbon sequestration. In the next 5 years, the carbon sequestration amount increases to 138.987 thousand tons. In the third 5-year interval, the sequestration amount declines to 104.234 thousand tons, followed by a further decrease to 92.479 thousand tons during the fourth 5-year span. Within the fifth 5-year period, carbon sequestration reaches 90.934 thousand tons, and in the last 5 years, it amounts to 87.864 thousand tons. The CO_2_ emissions in the project area change by the following amounts: − 10.6, − 12.446, − 9.334, − 8.281, − 8.143, and 7.868 thousand tons in the first, second, third, fourth, fifth, and sixth 5-year periods, respectively.

**Fig. 8:**
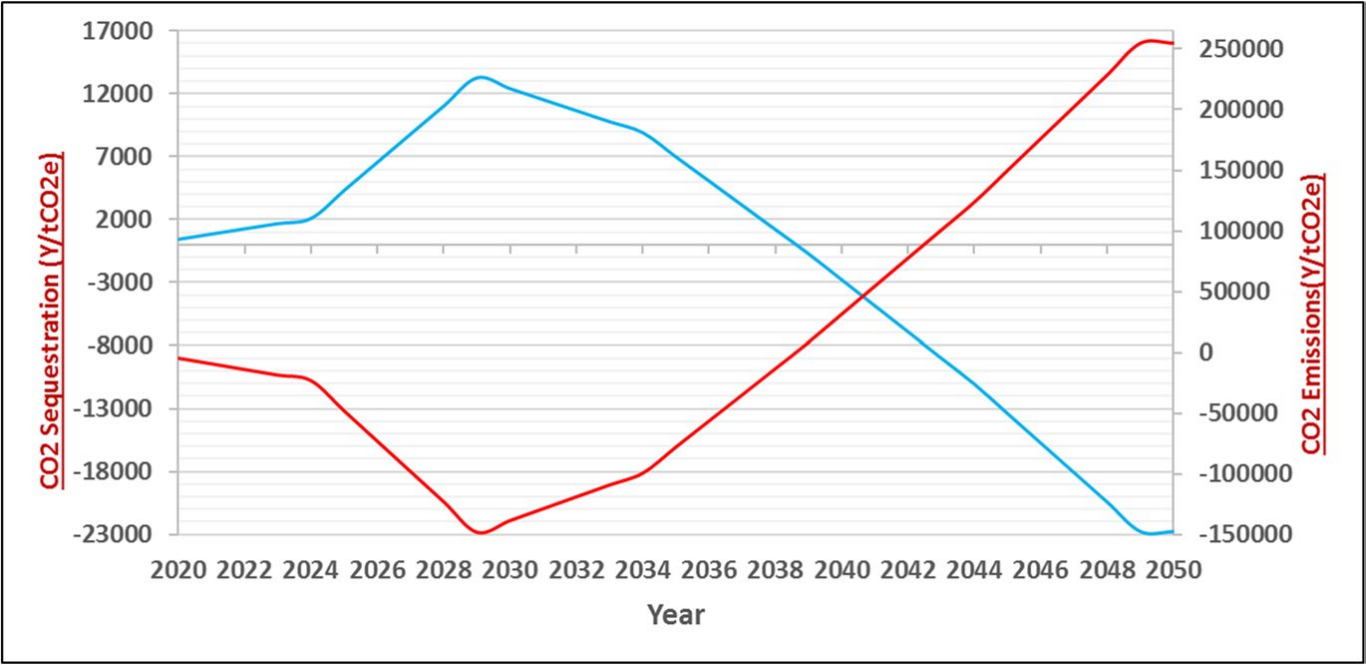
Cumulative diagram of CO2 emission and carbon sequestration in the leakage belt under BioCF methodology—red diagram is the cumulative amount of carbon sequestration due to forest to non-forest conversion in the leakage belt and the blue diagram is the cumulative CO2 emission in the leakage belt under BioCF methodology

Figure 8 depicts two completely different trends from 2020 to 2050. The minimum amount of CO_2_ emissions is equal to − 8.646 tons (2020), and the maximum amount is 254.5552 tons (2050). Affected by the lack of implementation of the BioCF method in the leakage belt, the cumulative carbon sequestration amount due to forest to non-forest conversion in the leakage belt (red curve) decreases from 2020 to 2027 but shows an upward trend from 2028 to 2050. The cumulative amount of CO_2_ emissions in the leakage belt (blue curve) increases from 2020 to 2027 but declines from 2028 to 2050. The amount of CO_2_ emissions in this period is approximately − 755 tons, and the amount of carbon sequestration due to the conversion of forests into non-forest areas is 8.432 tons. This change occurs because with an increase in forest to non-forest conversion, the percentage of CO_2_ emitted into the atmosphere decreases by the same amount.

According to Table 4, the actual amount of carbon emissions in the REDD scenario in the project area reduces from 108,030 tons per year in 2020 to 79,995 tons in 2050. Between 2020 and 2022, the trend of carbon emissions is projected to stabilize, undergoing no significant change. Between 2023 and 2027 (second 5-year period of project implementation), this trend increases again. Thus, in this period, this trend is increased by moving some processes and controlling the whole project area from nondeforestation. Gradually, beginning in the third 5-year period, the emissions decrease. This prevents the emission of a considerable amount of carbon into the atmosphere.

**Table 4:** The amount of actual carbon emissions under the project implementation. (A) CO2 emissions in the BioCF project area over the next 30 years; (B) actual carbon emissions in the REDD project area + CO2 emissions due to relocation of some deforestation activities under the REDD project

Year	A tCO2e	B tCO2e	Year	A tCO2e	B tCO2e	Year	A tCO2e	B tCO2e
2020	108,030	51,062	**2030**	94,900	78,010	**2040**	82,791	68,056
2021	108,030	51,062	**2031**	94,900	78,010	**2041**	82,791	68,056
2022	108,030	51,062	**2032**	94,900	78,010	**2042**	82,791	68,056
2023	126,541	78,015	**2033**	84,198	69,213	**2043**	79,995	65,759
2024	126,541	78,015	**2034**	84,198	69,213	**2044**	79,995	65,759
2025	126,541	78,015	**2035**	84,198	69,213	**2045**	79,995	65,759
2026	126,541	78,015	**2036**	84,198	69,213	**2046**	79,995	65,759
2027	126,541	78,015	**2037**	84,198	69,213	**2050**	79,995	65,759
2028	94,900	78,010	**2038**	82,791	68,056			
2029	94,900	78,010	**2039**	82,791	68,056			

According to Table 5, which shows the CO_2_ emission reduction due to the relocation of some deforestation activities in the project area in the REDD scenario, the average amount of CO_2_ emissions in the project area over the first 5 years (2020–2022) is 149,082 tons. In the second 5-year period (2023–2027), the average amount of CO_2_ emissions in the project area is 476,242 tons; in the third 5-year period (2027–2032), 855,850 tons; in the fourth 5-year period (2033–2037), 1,209,765 tons; in the fifth 5-year period (2038–2042), 1,543,179 tons; in the sixth 5-year period (2043–2050), 1,867,633 tons.

**Table 5:** Reduction of CO2 emissions due to relocation of some deforestation activities in the project area under REDD scenario. (A) CO2 emission rate due to relocation of some deforestation activities under the project; (B) CO2 emissions in the leakage belt

Year	A tCO2e	B tCO2e	Year	A tCO2e	B tCO2e	Year	A tCO2e	B tCO2e
2020	149,082	64,818	**2030**	855,850	263,041	**2040**	1,543,179	348,957
2021	198,776	86,424	**2031**	931,770	272,531	**2041**	1,609,412	357,236
2022	248,470	108,030	**2032**	1,007,690	282,021	**2042**	1,675,644	365,515
2023	324,394	133,338	**2033**	1,075,048	290,441	**2043**	1,739,641	373,515
2024	400,318	158,647	**2034**	1,142,406	298,860	**2044**	1,803,637	381,514
2025	476,243	183,955	**2035**	1,209,765	307,280	**2045**	1,867,633	389,514
2026	552,167	209,263	**2036**	1,277,123	315,700	**2046**	1,931,630	397,513
2027	628,092	234,571	**2037**	1,344,481	324,120	**2050**	1,995,626	405,513
2028	704,011	244,061	**2038**	1,410,714	332,399			
2029	779,931	253,551	**2039**	1,476,947	340,678			

As seen in Table 5, the average CO_2_ emission reduction in the leakage belt in the REDD scenario is 64,818 tons in the first 5 years (2020–2022), 183,954 tons in the second 5-year period (2023–2027), 263,041 tons in the third 5-year period (2027–2032), 307,280 tons in the fourth 5-year period (2033–2037), 348,957 tons in the fifth 5-year period (2038–2042), and 389,813 tons in the sixth 5-year period (2043–2050).

Table 6 shows that the amount of inhibited CO_2_ emissions increases from 36,730 tons in 2020 to 481,134 tons in 2050, which is a significant amount compared with the amount before project implementation. This process will promote the move of the area toward reforestation, biodiversity conservation, and the implementation of Kyoto Protocol policies, thus paving the way for a sustainable (a sustainable process that long-term health and resilience of ecosystems, reduces greenhouse gas emissions, and supports the well-being of communities), clean process (in the context of the Kyoto Protocol, a “clean process” may involve actions that reduce or mitigate greenhouse gas emissions, such as transitioning to renewable energy sources, improving energy efficiency, and implementing carbon capture and storage technologies).

**Table 6:** The amount of tCO2e inhibited in the atmosphere during the implementation of the REDD project. (A) CO2 emissions in the project area under the BioCF methodology over the next 30 years; (B) actual carbon emissions in the project area under the REDD scenario

Year	A tCO2e	B tCO2e	Year	A tCO2e	B tCO2e	Year	A tCO2e	B tCO2e
2020	324,091	110,191	**2030**	1,457,553	338,662	**2040**	2,316,714	424,578
2021	432,121	146,921	**2031**	1,552,453	348,152	**2041**	2,399,505	432,857
2022	540,151	183,651	**2032**	1,647,352	357,642	**2042**	2,482,296	441,136
2023	666,692	208,960	**2033**	1,731,550	366,062	**2043**	2,562,291	449,136
2024	793,233	234,268	**2034**	1,815,748	374,482	**2044**	2,642,286	457,135
2025	919,773	259,576	**2035**	1,899,946	382,901	**2045**	2,722,282	465,135
2026	1,046,314	284,884	**2036**	1,984,144	391,321	**2046**	2,802,277	473,134
2027	1,172,855	310,192	**2037**	2,068,342	399,741	**2050**	2,882,273	481,134
2028	1,267,754	319,682	**2038**	2,151,133	408,020			
2029	1,362,654	329,172	**2039**	2,233,923	416,299			

## Discussion

In this study, we aimed to assess the impact of implementing REDD on deforestation patterns and GHG emissions. Deforestation is the permanent removal of forests, whereas forest degradation refers to declines in forest quality and vitality (Houghton, 2012). Monitoring these two phenomena necessitate distinct methodologies and considerations. Our study focuses solely on deforestation while acknowledging the need for future investigations of the effects of forest degradation. Our methodology begins with the generation of precise multitemporal LULC maps. To accomplish this, we used innovative techniques leveraging time-series satellite imagery, which allowed us to extract spectral–temporal features. These features were then utilized to train an RF model for the production of LULC maps. We evaluated the accuracy of the generated LULC maps, and the findings affirmed their suitability for our subsequent analyses. An RF model was selected in this study because of its established superiority over alternative classification methods, including maximum likelihood methods, support vector machines, and neural networks, as validated by prior investigations (Adugna et al., 2022; Kamal et al., 2022).

Change detection analysis provided valuable insights into forest cover dynamics. Overall, we found a net increase of 8251 ha in forest cover from 1985 to 2020. We observed two distinct trends: a decline in forest cover from 1985 to 2000 and an increase from 2000 to 2020 (Fig. 3). The highest rate of forest recovery (8.91%) occurred between 2000 and 2010. By contrast, the 1990–1995 period exhibited the most extensive deforestation, showing a loss of − 9.87% of forested areas. The observed decline in forest cover during the period from 1985 to 2000 raises questions about the potential drivers of deforestation during this timeframe. Investigating the socio-economic, land-use, and policy factors influencing this particular period may offer valuable insights into the drivers of deforestation and inform targeted conservation efforts. Nasiri et al., (2023a, b) employed a modeling approach to explore the factors influencing deforestation and forest recovery in the Hyrcanian forests. Their findings highlighted that population growth, urbanization, and climate change emerged as particularly influential factors contributing to deforestation in the Hyrcanian forests. Also grazing and tourism activities are the main drivers of deforestation in the Hyrcanian forests (Jahanifar et al., 2020; Shooshtari et al., 2018).

Grazing substantially affects deforestation. Directly, it induces forest removal or degradation as animals exceed the land’s regenerative capacity by consuming vegetation. Indirectly, grazing contributes to deforestation through forest clearance for pasture or feed crop cultivation (Erb et al., 2016; Godde et al., 2018). Moreover, trampling and soil compaction resulting from grazing activities intensify soil erosion and degradation, impeding forest regeneration (Ligate et al., 2018). Tourism activities can contribute to deforestation through infrastructure development, land-use changes, unsustainable resource consumption, forest fragmentation, and illegal activities (Nasiri et al., 2018). However, well-managed ecotourism initiatives can promote forest conservation and provide alternative livelihoods. Therefore, sustainable practices should be adopted, local communities should be engaged, and regulations should be enforced to ensure that tourism benefits forests and minimizes deforestation. Conversely, the period from 2000 to 2020 stands out for its positive trajectory, showing a significant increase in forest cover. Understanding the factors contributing to this resurgence is crucial for both conservation and sustainable land management practices. The observed positive changes suggested a transition from unsustainable forest management practices to more sustainable approaches or the establishment of a balanced utilization of forests by local communities. According to Nasiri et al., (2023a, b), the implementation of the Forest Rest Plan (FRP) as a conservation policy in the Hyrcanian forest has shown promise in arresting deforestation trends and fostering forest recovery, particularly in regions with high levels of protection and ongoing restoration initiatives.

Regarding the deforestation trend in the leakage belt and the project area under the BioCF method, the results showed varying averages across different periods. In 2020–2022, the leakage belt (project area) had an average deforestation amount of − 8.28 ha (211.48 ha). The observed increase or decrease in GHG emissions within the leakage belt and the project area corresponded to the presence or absence of prevention measures. These emissions have direct implications for regional biomass dynamics and the potential extinction of valuable animal and plant species. Additionally, these emissions contribute to the exacerbation or mitigation of climate change impacts in a reciprocal manner.

In summary, REDD projects incentivize forest conservation and emission reduction to mitigate climate change; they can also significantly slow the extinction rate of forest species (Busch et al., 2011). This includes ensuring that local people and communities are informed about such projects and are consulted in the decision-making process; project outcomes are also rigorously monitored. Additionally, REDD projects should be integrated with other activities, such as sustainable land-use management and biodiversity conservation efforts. The conservation and restoration of natural forests can be highly effective (Milne et al., 2019). The Iranian Parliament endorsed the Forest Rest Plan in 2016 within this framework. The plan prohibits all commercial and industrial wood harvesting in the Hyrcanian forests to mitigate their deforestation and enhance their vegetation, resilience, and productivity. The successful implementation of this conservation plan and the implementation of REDD in the Hyrcanian forests are anticipated to decrease the GHG emissions of the forests.

As shown by 40 voluntary projects across nine countries, REDD projects effectively decrease deforestation and forest degradation relative to the first 5 years of their implementation (Guizar-Coutiño et al., 2022). Within the Hyrcanian forests, as evidenced by a study conducted in the Nowshahr and Noor forests (Parsamehr et al., 2023), the results of REDD baseline modeling reveal a projected release of 827,591.5 metric tons of carbon dioxide equivalent (tCO2e) over the next 30 years (2014–2044). However, the implementation of the REDD project in these areas is anticipated to significantly curtail this release, preventing the emission of approximately 584,056.38 tCO2e during the same period. In another study conducted in Iran, Delpasand et al. (2022) assessed the transformations in the Zagros Forest cover over a 20-year period and forecasted the potential outcomes of implementing the REDD + project in the forthcoming two decades. The study reveals a loss of 37,809 hectares of forest cover during the past two decades, with an associated increase in greenhouse gas emissions. However, promisingly, the implementation of the REDD + project is anticipated to avert the release of approximately 1,714,534.13 metric tons of carbon dioxide equivalent (tCO2e), offering a significant positive impact on emission reduction and forest conservation in the region. Therefore, similar to previous studies in Iran, our findings provide valuable insights into the potential impact of implementing REDD projects in Iranian forests. This indicates that the REDD initiative has the potential to play a crucial role in mitigating carbon emissions and promoting sustainable forest management in the Hyrcanian. Furthermore, the implementation of the BioCF method in the leakage belt improved the carbon sequestration rate due to the conversion of forests into non-forest areas and the reduction of CO_2_ emissions. Therefore, the BioCF method should be used more efficiently to reduce the negative impacts of deforestation on the environment.

However, this method has limitations and problems. Major obstacles to REDD implementation stem from the need for substantial international commitment to climate change reduction and increased carbon financing (Sunderlin et al., 2015). The complexity and cost of measuring and monitoring forest carbon stocks may limit the efficacy of REDD initiatives. REDD projects require significant funds, which may be difficult to obtain for many countries. Moreover, the success of REDD projects may be constrained by the uncertainty of their long-term financial viability. Another hindrance is the possible lack of an overall reduction in emissions. For instance, if a REDD project slows down deforestation in one location, leakage (the movement of emissions from one area to another) can increase in another. Overall, although REDD can be an essential tool against climate change, these limitations should be addressed to maximize its benefits.

**Fig. 9:**
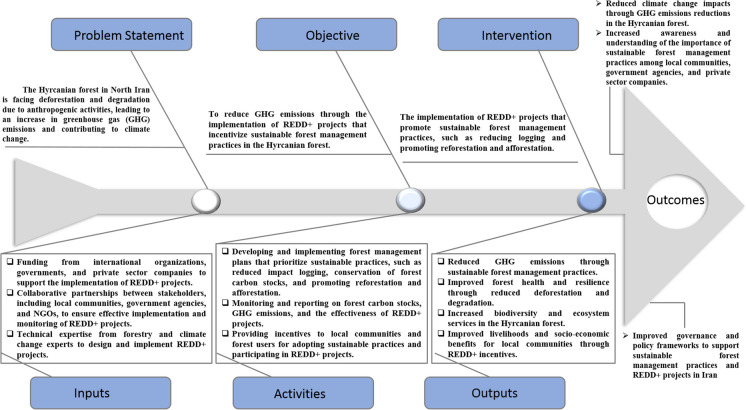
Climate change mitigation through the implementation of REDD + projects

The conceptual model in Fig. 9 shows climate change mitigation through the implementation of REDD projects that incentivize sustainable forest management practices in the Hyrcanian forests, north Iran. The conceptual model of REDD projects is based on several fundamental principles, such as an understanding of the importance of forests for mitigating climate change, the need to address the root causes of deforestation and forest degradation, and the significance of involving local communities in project planning and implementation. The need to address the underlying causes of deforestation and forest degradation is a critical element of this conceptual model. This model also emphasizes the importance of involving local populations in the planning and execution of REDD programs. By reducing GHG emissions and promoting biodiversity and ecosystem services, the approach presented in Fig. 9 can contribute to the global effort to address climate change while benefiting local communities and improving forest health and resilience.

Finally, based on our findings, in order to safeguard the Hyrcanian forests through the REDD project, we propose several practical recommendations:

1. Strengthen monitoring and surveillance systems to efficiently identify and deter illegal logging, encroachments, and other activities that contribute to deforestation.
2. Employ advanced technologies, such as satellite imagery and remote sensing, for real-time monitoring of changes in forest cover.
3. Engage local communities in the decision-making process and the implementation of conservation measures. Their active involvement can significantly contribute to sustainable forest management.
4. Execute community-based initiatives that offer alternative livelihoods and economic opportunities, reducing reliance on forest resources.
5. Allocate resources for reforestation and afforestation projects aimed at restoring degraded areas and enhancing overall forest cover.
6. Prioritize native and endemic species in reforestation efforts to preserve the ecological diversity of the Hyrcanian forests.

## Conclusion

The primary objective of this study is to combat the pressing issue of global warming by strategically targeting the reduction of GHG emissions. Specifically, it delves into the potential impact of executing a REDD project within the protected area of Kojoor, situated in the Hyrcanian forests of northern Iran. By focusing on the implementation of the REDD framework, this research endeavors to gauge its efficacy in curbing GHG emissions within the Hyrcanian forests. Despite its regional focus on Kojoor, the implications and outcomes derived from this study can be extrapolated to benefit other areas within the Hyrcanian forests and similar ecological sites globally. The study’s findings underscore a crucial insight: in the absence of REDD implementation, the trajectory would lead to deforestation, consequently resulting in the release of significant amounts of CO2-equivalent emissions into the atmosphere. However, the implementation of REDD emerges as a pivotal intervention capable of counteracting this detrimental trend. It effectively mitigates emissions, preventing the release of CO2-equivalent emissions into the upper atmosphere. This research not only highlights the potential catastrophic consequences of deforestation but also showcases the substantial positive impact that strategic initiatives like REDD can have in preserving these vital ecosystems and mitigating the adverse effects of climate change on a broader scale. To harness the advantages of REDD implementation effectively, it is crucial that national GHG emission reduction programs and forest management strategies are intricately designed to align with international agreements like REDD. This involves creating strategies that bolster forest carbon sequestration or prevent emissions, accomplished through comprehensive forest conservation plans, reforestation efforts, and the provision of socio-economic support. A main limitation of studies that use historical LULC data to predict future LULCCs to assess the impact of REDD projects on climate change and GHG emissions is the inherent uncertainty associated with these predictions. Although historical trends can provide insights, future land-use dynamics are influenced by numerous unpredictable factors, such as changes in economic conditions, policy shifts, technological advancements, and social preferences. These uncertainties can affect the accuracy of projected LULCCs and hence the estimation of GHG emissions and the effectiveness of REDD projects in mitigating climate change. Additionally, the complexity of land-use decision-making processes, which involve multiple stakeholders with varying motivations and priorities, adds another layer of uncertainty to these predictions. Furthermore, the accuracy of LULCC data and the quality of the remote sensing methods used for prediction can introduce uncertainties and errors in the analysis. These limitations highlight the need for robust sensitivity analysis that incorporates various scenarios and considers multiple influencing factors, which will improve the reliability and robustness of predictions and assessments of the effects of REDD projects on climate change and GHG emissions**.**

## Data Availability

The authors declare that data supporting the findings of this study are available within this article and all simulation datasets generated and/or analyzed during the current study are available from the corresponding author on reasonable request.
